# Epidemiology and Characterization of Immunological Disorders in Children Diagnosed With Pneumococcal Invasive Disease

**DOI:** 10.1155/ijpe/9940233

**Published:** 2026-09-15

**Authors:** Chen Shahar, Gilat Livni, Nufar Marcus, Yehonatan Pasternak

**Affiliations:** ^1^ Department of Pediatrics A, Schneider Children′s Medical Center, Petah Tiqva, Israel; ^2^ Gray Faculty of Medical & Health Sciences, Tel Aviv University, Tel Aviv, Israel, tau.ac.il; ^3^ Infectious Diseases Unit, Schneider Children′s Medical Center, Petah Tiqva, Israel; ^4^ Kipper Institution for Clinical Immunology and Allergy, Schneider Children′s Medical Center, Petah Tiqva, Israel

**Keywords:** hyposplenism, immunological disorders, invasive pneumococcal disease, pneumococcus

## Abstract

**Aim:**

A substantial proportion of children hospitalized with invasive pneumococcal infection are subjected to immune evaluations, to detect immunological disorders (ID). The literature lacks thorough analysis of the necessity and effectiveness of these evaluations for previously healthy children with such infections. We aimed to examine the occurrence and characteristics of ID in children hospitalized with invasive pneumococcal disease (IPD), and to assess when immune investigations are most effective.

**Methods:**

In a retrospective study, we examined the immunological assessments and outcomes of children with IPD admitted during 2010–2023 to Schneider Children′s Medical Center.

**Results:**

Out of 219 children with IPD, 144 previously healthy patients were included, with a mean age of 2.2 years and an average hospital stay of 9 days. Immunological evaluations, defined as any immune‐related assessment, were performed in 94 patients (65.3%) and were not uniform across the cohort. ID were identified in 15 of 144 children (10.4%), corresponding to 15 of 94 evaluated patients (15.9%). Functional hyposplenism was the most frequent finding. No clinical or laboratory features reliably distinguished those with ID.

**Conclusion:**

Immunological evaluation should be considered in previously healthy children diagnosed with IPD. Functional hyposplenism should be considered when routine immunological evaluation is unrevealing; however, the role of universal spleen scintigraphy requires further prospective evaluation.

**Summary**

• The need for routine immune evaluations in previously healthy children hospitalized with invasive pneumococcal disease remains unclear.
• In a retrospective study, we found that 10.4% of children with invasive pneumococcal disease and 15.9% of evaluated patients were diagnosed with immunological disorders, with functional hyposplenism being the most frequent finding.
• These results support considering immunological assessment in previously healthy children presenting with IPD. Spleen scintigraphy may be considered in selected patients, especially when routine immunological evaluation is unrevealing, but our data do not establish the diagnostic yield of universal scintigraphy screening.

## 1. Introduction

*Streptococcus pneumoniae* is a gram‐positive bacterium with over 90 known subgroups (serotypes), most of which are part of the natural flora of the nasopharynx. Fewer than 20 subtypes are responsible for more than 90% of significant infections caused by the bacterium [1]. Invasive pneumococcal disease (IPD) is defined as the growth of bacterium in a culture from a sterile site such as blood, cerebrospinal fluid, pleural fluid, or joint fluid; bacteremia is the most common discharge diagnosis [2]. Several factors are known to increase the risk of pneumococcal infection, including ID such as inborn errors of immunity (IEI), which may involve dysfunction of the humoral, cellular, or complement systems. Other notable risk factors include prematurity, malnutrition, and HIV infection [3]. The proportion of children reported to have primary immune deficiency due to significant pneumococcal infection ranges from 1.3% to 10.4% [3]. Previous studies have reported that defects in the humoral arm of the immune system are the most common IEI associated with IPD [4]. In contrast, in our cohort the most frequently identified abnormality was functional hyposplenism.

Notably, some studies that characterized IPD reported that none of those with bacteremia had immune deficiency [3, 5]. However, when bacteremia was included under a broad spectrum of sepsis, the likelihood was higher of being diagnosed with immunodeficiency in the context of sepsis [6]. A substantial proportion of children hospitalized because of IPD undergo immune evaluation to diagnose rare instances of immune deficiency. However, the literature is sparse regarding the required scope and effectiveness of immune evaluation in healthy children who present with IPD.

In 2009, the 13‐valent PCV vaccine became part of Israel′s routine vaccination schedule. The implementation of the vaccination policy for conjugated pneumococcal vaccine has led to a significant decrease in morbidity, including bacteremia [1, 7]. However, it remains unclear whether IPD caused by pneumococci strains included in the vaccine carries a higher risk for ID.

In this study, we examined the occurrence and characteristics of ID in children hospitalized with IPD. Additionally, we aimed to identify when immune investigations are most effective and to establish potential approaches for investigating IPD.

## 2. Methods

This retrospective study was conducted at Schneider Children′s Medical Center of Israel, a tertiary, university‐affiliated children′s hospital. The hospital microbiology database was examined for all the patients aged 0–18 years who were hospitalized in one of the pediatric departments and diagnosed with IPD during 2010–2023.

IPD was defined by bacterial growth in cultures from sterile sites such as blood, cerebrospinal fluid, pleural fluid, and joint fluid. Patients with pneumococcal pneumonia were included only if they also had a positive blood culture or growth in pleural fluid. Exclusion criteria were the diagnosis of a chronic disease (e.g., cardiac malformation, and chronic pulmonary disease) or a prior diagnosis of immunosuppression, either congenital or acquired (malignancy, chemotherapy or biological therapy, chronic corticosteroid therapy, etc.). Immunological abnormalities were interpreted according to standard clinical immunology practice and, when applicable, in the context of the International Union of Immunological Societies (IUIS) classification of IEI [8]. Demographic data (gender, age, admission/discharge dates, and infection site) and laboratory data (complete blood count, inflammatory markers, and electrolytes) were collected. Immune evaluations during hospitalization or follow‐ups were also reviewed.

For the purposes of this retrospective study, “immunological evaluation” was defined as any immune‐related assessment performed during hospitalization or follow‐up, including one or more of the following: immunoglobulin concentrations, IgG subclasses, complement testing, vaccine responses, lymphocyte immunophenotyping, blood smear examination, spleen scintigraphy, or genetic evaluation. The extent of evaluation was not standardized and was determined by the treating physicians and immunologists according to clinical judgment, test availability, age, infection phenotype, disease severity, and follow‐up attendance.

Continuous variables are presented as means and standard deviations (SDs), and categorical variables as counts and percentages. We used one‐way ANOVA and Fisher′s exact test for group comparisons, facilitated by the R summary package.

This retrospective study was approved by the Rabin Medical Center Institutional Review Board (Helsinki Committee), approval number RMC‐0641‐22. Because of the retrospective design and the use of de‐identified clinical data, the requirement for written informed consent from participants or their legal guardians was waived by the committee.

## 3. Results

### 3.1. Patients′ Characteristics

In total, 219 children with IPD were identified, all were confirmed by a positive culture from a sterile site. After applying the exclusion criteria, the study included 144 previously healthy patients, diagnosed with IPD. Their mean age was 2.2 years (SD 4.95). The mean hospital stay was 9 days (SD 5). The most common discharge diagnosis was bacteremia, followed by meningitis. Blood cultures were obtained for all patients prior to initiation of parenteral antibiotic therapy. Most patients did not receive oral antibiotics before blood culture acquisition (confirmed in 108 patients), whereas 4 patients had documented prior oral antibiotic treatment.

Demographics and clinical characteristics are shown in Table 1. Discharge diagnoses other than bacteremia were classified into two categories: those with bacteremia and those without bacteremia.

**Table 1:** Demographic and clinical characteristics of children hospitalized with invasive pneumococcal disease.

Characteristic		Value (*n* = 144)	
Male sex (*n*, %)		90 (62.5)	
Age (years), mean (SD)		2.2 (5.0)	
Ethnicity (Jewish/Arab, %)		128 (88.9)/16 (11.1)	
Hospitalization duration (days), mean (SD)		9 (5)	
Discharge diagnosis (%)	Bacteremia	97 (67.4)	
		With bacteremia (%)	Without bacteremia (%)
	Meningitis	17 (11.8)	5 (3.5)
	Pleuropneumonia	4 (2.8)	9 (6.3)
	Septic arthritis	0	8 (5.6)
	Peritonitis	0	1 (0.7)
	Mastoiditis	3 (2.1)	0
	Total	24 (16.7)	23 (16.0)

Abbreviation: SD, standard deviation.

### 3.2. Immunological Evaluation

Immunological evaluation, defined as any immune‐related assessment, was performed in 94 patients (65.3%). The extent of evaluation varied substantially and was not based on a uniform protocol (Table 2). Among the 94 evaluated patients, immune‐related testing was performed in selected combinations according to clinical judgment and follow‐up availability, and included immunoglobulin concentrations, IgG subclasses in a subset of patients, complement evaluation, vaccine response assessment, lymphocyte immunophenotyping, blood smear examination, spleen scintigraphy, and genetic evaluation. Complement evaluation was performed in 62 patients (43.1%), primarily through measurement of C3 and C4 concentrations as an initial screening. Additional complement testing, including CH50 and extended complement components (C1–C9), was performed in selected patients based on clinical indication. Only 23 of 144 patients (16.0%) had follow‐up visits at the immunology clinic.

**Table 2:** Immune evaluation among patients diagnosed with invasive pneumococcal disease.

Immunologic evaluation performed		Tested number (%)	Abnormal result (% of tested)
Hematologic evaluation	Complete blood count with differential (%)	144 (100)	
	Blood smear	17 (11.8)	1^a^ (5.9)
Inflammatory markers	C‐reactive protein	144 (100)	135 (93.8)
	Erythrocyte sedimentation rate	24 (16.6)	23 (95.8)
Humoral evaluation	IgG, IgA, and IgM	94 (65.2)	2 (2.1)
	IgG Subclasses	25 (17.3)	2 (8)
	Vaccination responses	43 (29.2)	2 (4.7)
Complement evaluation	C3, C4	62 (43.1)	NA
	CH50	49 (34)	NA
	C1–9	13 (9)	NA
Imaging	Abdominal ultrasound	26 (18.0)	7 (26.9)
	Spleen scintigraphy	15 (10.4)	13 (86.7)

^a^Howell–Jolly bodies.

Following the detailed evaluation described above, 15 children (10.4%) were diagnosed with ID, of whom 13 had functional hyposplenism. One of the latter also presented with neutropenia. Of the two patients without functional hyposplenism, one had hypogammaglobulinemia associated with common variable immunodeficiency and was diagnosed with specific antibody deficiency. The other one had immunoglobulin A deficiency. Only one of the patients with hyposplenism had Howell–Jolly bodies detected on blood smear.

Thus, immunological disorders were diagnosed in 15 of the 144 previously healthy children with IPD (10.4%). Since some form of immunological evaluation was performed in 94 children (65.3%), the diagnostic yield among evaluated patients was 15 of 94 (15.9%). The proportion calculated among the entire cohort should therefore be interpreted as a conservative estimate, as children who did not undergo immunological assessment were assumed not to have an identified immunological disorder.

Fifteen patients underwent spleen scintigraphy, of whom 13 were found to have a hypofunctional spleen. Spleen scintigraphy was performed after resolution of the acute infection. All patients with abnormal scintigraphy had anatomically present spleens, excluding congenital asplenia and suggesting functional hyposplenism rather than infection‐related transient splenic dysfunction. The other two patients also had abnormal results on spleen scintigraphy, yet because of lack of regular follow up, they were not considered immunocompromised.

Spleen scintigraphy was not performed systematically in all evaluated children. During the study period, referral for spleen scintigraphy was based on the treating immunologist′s clinical judgment, mainly in children with pneumococcal bacteremia in whom the initial immunological workup did not reveal an alternative explanation, or when functional hyposplenism was clinically suspected. Therefore, the subgroup undergoing scintigraphy represents a selected population rather than a predefined screening cohort.

Genetic evaluation was performed in four patients. Only one child, 11 years old, diagnosed with common variable immunodeficiency, underwent a dedicated immunodeficiency gene panel, which identified a variant of uncertain significance (VUS) in the VAV1 gene.

Serotype tracking in patients with ID identified various bacterial serotypes: five of 12F, two of 10B, and one each of 16F, 18C, 15B, A19 and 38. One patient presented with an untypable serotype, and for two, the serotype could not be determined. Among the 12 patients in whom the serotype was identified, only two (16.7%) corresponded to serotypes included in the PCV‐13 vaccination program in Israel (19A and 18C).

### 3.3. Risk Factors

Laboratory test characteristics for each patient with IPD were examined to identify risk factors for ID. Gender and ethnicity distributions, age at admission, and the site of the positive culture did not differ significantly between the groups (Table 3). Bacteremia presented in all the patients with and in 82.2% of the patients without ID. Immunological disorders were identified exclusively among children with bacteremia (15/121, 12.4%), whereas no cases were detected among patients without bacteremia (0/23) (Table 1).

**Table 3:** Comparison of demographic and clinical characteristics between patients with and without a diagnosis of immunological disorder.

Characteristics	No IEI *N* = 129	Any IEI *N* = 15	*p*
Gender (male, %)	79 (61.2)	11 (73.3)	0.41
Ethnicity (Arab, %)	13 (16.4)	3 (20.0)	0.37
Age at admission (years, SD)	2.29 (2.9)	1.57 (2.8)	0.36
Site of positive culture, *n* (%)			
Only blood stream	87 (67.4)	10 (66.7)	
CSF	19 (14.7)	3 (20.0)	
Pleural effusion	12 (9.3)	1 (6.7)	
Joint	8 (6.2)	0 (0.0)	
Mastoid	2 (1.6)	1 (6.7)	
Peritoneal	1 (0.8)	0 (0.0)	
Bacteremia present	106 (82.2)	15 (100)	

Abbreviations: CSF, cerebrospinal fluid; SD, standard deviation.

Among the 23 children with nonbacteremic IPD, 16 (69.6%) underwent some form of immunological evaluation, and no definitive immunological disorder was diagnosed. This evaluation rate was comparable to that observed in the overall cohort, in which 94 of 144 children (65.3%) underwent some form of immunological assessment.

## 4. Discussion

In this retrospective cohort, immunological disorders were diagnosed in 10.4% of all previously healthy children with IPD and in 15.9% of those who underwent some form of immunological evaluation. Because the immunological workup was selective and nonuniform, these figures should be interpreted as the observed diagnostic yield in our clinical setting rather than the true incidence of immunological disorders among all children with IPD. This is at the higher end of the range reported previously [5]. The substantial proportion may be attributed to our setting at the largest children′s hospital in Israel, and to the accessibility of the immunological clinic within the hospital. The result underscores the importance of considering an immune evaluation for children with IPD.

Although individual IEIs are rare, collectively IEIs are not, and they represent a substantial health burden. Indeed, the incidence of IEIs in the United States was reported as 6 per 10,000 people [9]. It is reasonable to assume that the prevalence in Israel may be higher, because of the higher rates of consanguinity in certain populations.

The most common ID identified in our study was hyposplenism, or hypofunction of the spleen. Specifically, this represented functional hyposplenism, without congenital asplenia or evidence of infection‐related transient splenic dysfunction. This finding has significant implications for therapy and highlights the critical need for early detection, given the spleen′s essential role in combating encapsulated bacteria [10, 11]. Additionally, patients with this diagnosis may benefit from vaccination against common infectious organisms [10–12]. Some individuals with functional hyposplenism were reported to improve or even reach normalization over time [13]. This underscores the importance of ongoing immunological follow‐up. The high proportion of abnormal spleen scintigraphy findings among tested children suggests that functional hyposplenism should be considered in children with pneumococcal bacteremia, particularly when routine immunological evaluation does not reveal an alternative explanation. However, because spleen scintigraphy was performed in only 15 patients according to clinical judgment rather than systematically, our findings cannot determine the yield or cost‐effectiveness of universal spleen scintigraphy screening. Prospective studies using predefined criteria are needed to determine which children with IPD would benefit most from splenic functional imaging.

Notably, some children with hyposplenism did not have Howell–Jolly bodies in their blood smear. As shown previously [13], a negative blood smear does not rule out a hypofunctional spleen or hyposplenism. In the future, the diagnosis of hyposplenism may become more accessible through wider use of flow cytometry‐based B cell profiling. Quantifying IgM memory B cells offers a sensitive and practical marker of splenic dysfunction [14]. This approach may enable earlier detection and more tailored clinical management.

In our cohort, all children diagnosed with an immunological disorder presented with bacteremia, whereas no definitive immunological disorder was diagnosed among patients with nonbacteremic IPD. This corresponds to a diagnostic yield of 12.4% among bacteremic cases and 0% among nonbacteremic cases. Among the 23 children with nonbacteremic IPD, 16 (69.6%) underwent some form of immunological evaluation, a proportion comparable to that observed in the overall cohort (94/144, 65.3%).

Although the sample size of the nonbacteremic group was limited, and the extent of immunological evaluation was not uniform, the comparable evaluation rate suggests that under‐evaluation of this subgroup is unlikely to fully explain the absence of diagnosed immunological disorders. These findings suggest that pneumococcal bacteremia may represent the clinical phenotype most strongly associated with underlying immunological dysfunction in previously healthy children with IPD. Accordingly, although immunological evaluation appears justified in cases of pneumococcal bacteremia, our data do not provide strong support for routine investigation in all isolated nonbacteremic presentations. Nevertheless, larger prospective studies are warranted to confirm this observation. This finding differs from the conclusion of a previous study [5]. The discrepancy may be related to differences in cohort composition, including variation in the proportion and clinical characteristics of patients with bacteremia. A second explanation is the underrepresentation of sepsis and overrepresentation of meningitis in their study, as most of the patients were hospitalized in the intensive care unit. In contrast to studies that reported limited benefit from such investigations in pediatric patients with bacteremia [5, 15], our findings revealed instances of hyposplenism or splenic hypofunction in these children. This discrepancy may be attributed to the earlier studies not utilizing spleen scintigraphy or relying solely on blood smears, which could overlook splenic abnormalities in the absence of Howell–Jolly bodies.

The absence of identifiable risk factors complicates decisions regarding when to pursue immunological investigations. Currently, there are no established guidelines for the timing of immunological evaluations in children with IPD. Recent work has proposed structured diagnostic approaches for evaluating children with severe pyogenic infections that may reflect underlying immunological disorders [16]. These frameworks emphasize that invasive bacterial infections may represent the first clinical manifestation of immune dysfunction and support the rationale for considering immunological evaluation in patients presenting with IPD. In particular, subtle and often clinically unapparent abnormalities affecting innate immune pathways have increasingly been recognized as predisposing factors for pyogenic infections caused by encapsulated bacteria such as *S. pneumoniae* [17]. Moreover, emerging evidence suggests that susceptibility to respiratory infections may lie along a continuum ranging from common genetic variants to fully penetrant IEI, which may partly explain why clinical features alone did not reliably distinguish patients with underlying immune dysfunction in our study population [18]. Our findings suggest that evaluation for splenic dysfunction may be relevant in selected children with IPD, particularly those with pneumococcal bacteremia, together with the emerging role of genetic testing, which unfortunately was not systematically pursued in our cohort.

Vaccination status was available for most children diagnosed with immunological disorders. Eight of these children had received at least two doses of pneumococcal conjugate vaccine according to the national schedule. Four children had not received two doses, primarily because of young age or incomplete vaccination. For three children, vaccination status could not be verified. Importantly, serotype analysis demonstrated that only a minority of infections were caused by serotypes included in the PCV‐13 vaccine currently used in Israel [19]. Among the 12 patients with identified serotypes, only two (16.7%) corresponded to PCV‐13 serotypes (19A and 18C). This observation suggests that a substantial proportion of cases may not have been preventable with the currently implemented vaccine program. Newer higher‐valency pneumococcal conjugate vaccines, such as PCV‐20, may theoretically expand coverage; however, additional epidemiological studies are needed to determine their potential impact in this population. These findings further emphasize that invasive pneumococcal infection in previously healthy children should prompt consideration of an underlying immunological disorder regardless of vaccination status.

Our study has several limitations. Firstly, it is a retrospective analysis conducted at a single tertiary center, and this may have resulted in a high prevalence of immunodeficient children compared with other settings. This single‐center approach could limit the generalizability of the findings to other hospitals or geographic regions. In addition, humoral evaluation was not uniform across the cohort, and specific polysaccharide antibody deficiency cannot be excluded in all patients. Complement evaluation in our cohort primarily consisted of measuring C3 and C4 concentrations, and a comprehensive assessment of the complement pathways (e.g., C1, C2, or functional assays) was not systematically performed. Another limitation is the limited use of genetic testing. Genetic evaluation was performed in only four patients, and only one child underwent a dedicated immunodeficiency gene panel. This reflects the retrospective nature of the study, the long inclusion period from 2010 to 2023, evolving availability and clinical use of genetic testing, and the fact that genetic studies were performed according to clinical judgment rather than as part of a standardized protocol. Consequently, some monogenic IEI may have been overlooked, and the diagnosed conditions in this cohort should be interpreted primarily as clinically and functionally defined immunological disorders rather than genetically confirmed IEI.

Additionally, although the cohort was large and the study period was 14 years, the retrospective design precluded complete information and potentially introduced biases. A possible bias is that immunological evaluation was not performed in all patients and was not uniform among those who were evaluated. Although most children (94/144 [65.3%]) underwent some form of immunological testing, the evaluation was performed according to clinical judgment and may therefore introduce selection bias if patients with more severe presentations were preferentially investigated. However, even under the conservative assumption that all nonevaluated patients were immunologically normal, the minimum prevalence of immunological disorders in the cohort would remain 15 out of 144 patients (10.4%).

Despite the abovementioned limitations, the findings are valuable and underscore the importance of considering immunological evaluation in children with pneumococcal bacteremia, irrespective of whether the serotype is included in the vaccination program. Future studies should be conducted across multiple centers and consider the introduction of the new 20‐valent pneumococcal vaccine to better understand its impact on IPD and to guide vaccination strategies accordingly.

## 5. Conclusion

In conclusion, immunological disorders were identified in a clinically meaningful proportion of previously healthy children with IPD, particularly among those presenting with pneumococcal bacteremia. Because immunological evaluation was selective and nonuniform, the observed proportions should be interpreted as diagnostic yield rather than true incidence. Functional hyposplenism was the most frequent finding, suggesting that splenic dysfunction should be considered in selected children with pneumococcal bacteremia, especially when routine evaluation is unrevealing. Larger prospective studies using standardized immunological and genetic evaluation protocols are needed to define the optimal diagnostic approach for previously healthy children presenting with IPD.

## Funding

No funding was received for this manuscript.

## Conflicts of Interest

The authors declare no conflicts of interest.

## Data Availability

The data that support the findings of this study are available from the corresponding author upon reasonable request.
